# Efficacy of intra-articular injections of platelet-rich plasma as a symptom- and disease-modifying treatment for knee osteoarthritis - the RESTORE trial protocol

**DOI:** 10.1186/s12891-018-2205-5

**Published:** 2018-07-28

**Authors:** Kade L. Paterson, David J. Hunter, Ben R. Metcalf, Jillian Eyles, Vicky Duong, Jessica Kazsa, Yuanyuan Wang, Rachelle Buchbinder, Flavia Cicuttini, Andrew Forbes, Anthony Harris, Shirley P. Yu, Bing Hui Wang, David Connell, James Linklater, Kim L. Bennell

**Affiliations:** 1 Centre for Health, Exercise and Sports Medicine, Department of Physiotherapy, School of Health Sciences, Faculty of Medicine Dentistry & Health Sciences, The University of Melbourne, Melbourne, VIC Australia; 2 Rheumatology Department, Royal North Shore Hospital Australia and Institute of Bone and Joint Research, Kolling Institute, University of Sydney, Sydney, Australia; 3 Department of Epidemiology and Preventive Medicine, School of Public Health and Preventive Medicine, Monash University, Melbourne, Australia; 4 Monash Department of Clinical Epidemiology, Cabrini Institute, Melbourne, Australia; 5 Musculoskeletal Unit, Department of Epidemiology and Preventive Medicine, Monash University and Rheumatology Unit, Alfred Hospital, Melbourne, Australia; 6 Centre for Health Economics, Monash University, Melbourne, Australia; 7 Monash Centre of Cardiovascular Research & Education in Therapeutics, Department of Epidemiology and Preventive Medicine, Monash University, Melbourne, Australia; 8 Imaging at Olympic Park, Melbourne, Australia; 9 Castlereagh Imaging, Sydney, Australia

**Keywords:** Osteoarthritis, Knee, Cartilage, Pain, Platelet-rich plasma, Saline, Intra-articular injection, Clinical trial

## Abstract

**Background:**

Knee osteoarthritis (OA) causes substantial pain, physical dysfunction and impaired quality of life. There is no cure for knee OA, and for some people, the disease may involve progressive symptomatic and structural deterioration over time. Platelet-rich plasma (PRP) is a therapeutic agent that aims to address underlying biological processes responsible for OA pathogenesis. As such, it has the potential to improve both symptoms and joint structure. The aim of this clinical trial is to determine whether a series of injections of PRP into the knee joint will lead to a significantly greater reduction in knee pain, and less loss of medial tibial cartilage volume over 12 months when compared to a series of placebo saline injections in people with knee OA.

**Methods:**

This will be a two-group, superiority, randomised, participant-, interventionist- and assessor-blinded, placebo-controlled trial. Two hundred and eighty-eight participants aged over 50 years with painful knee OA and mild to moderate structural change on x-ray (Kellgren and Lawrence grade 2 and 3) will be randomly allocated to receive either three PRP injections or three normal saline injections into the knee joint at weekly intervals. The primary outcomes will be 12-month change in average overall knee pain severity (numeric rating scale) and medial tibial cartilage volume (magnetic resonance imaging (MRI)). Secondary outcomes include additional measures of knee pain and other symptoms, function in daily living and sport and recreation, quality of life, participant-perceived global ratings of change, and other MRI structural outcomes including meniscal and cartilage morphology, synovitis, effusion, bone marrow lesions and cartilage defects. A range of additional measures will be recorded, and a separate health economic evaluation will be performed.

**Discussion:**

The findings from this study will help determine whether PRP improves both clinical and structural knee OA outcomes over 12 months when compared to a series of placebo saline injections.

**Trial registration:**

Australian New Zealand Clinical Trials Registry reference: ACTRN12617000853347. Prospectively registered 9th of June 2017.

**Electronic supplementary material:**

The online version of this article (10.1186/s12891-018-2205-5) contains supplementary material, which is available to authorized users.

## Background

Osteoarthritis (OA) is a leading causes of global disability, and the number of people affected is anticipated to substantially increase over the coming decades [1]. Osteoarthritis at the knee joint, the most commonly involved lower limb site, can cause pain and physical dysfunction and impaired quality of life. There is no cure for knee OA, and to date, most research has focused on treatments to alleviate pain and prevent functional decline.

Recommended drug therapies (such as analgesics and anti-inflammatory agents) and non-drug therapies (such as exercise) have short-term clinical benefits, but effect sizes are small to moderate at best [2, 3]. Furthermore, drug therapies can have adverse events, while uptake and maintenance of exercise are often poor leading to lack of long-term benefit. Intra-articular therapies in clinical use for knee OA include glucocorticoids and hyaluronic acid (a viscosupplement). Intra-articular glucocorticoids are generally recommended [4–6], although not universally [7], and for short-term pain relief only given that benefits are limited to a few weeks [6, 8]. Furthermore, a recent clinical trial highlighted a potential small deleterious effect of repeated corticosteroid injections on knee joint cartilage [9]. Hyaluronic acid is controversial with most clinical guidelines advising against its use [4, 10], or providing an uncertain recommendation [6]. As knee OA is a chronic disease, with both symptoms and structural deterioration drivers for surgical joint replacement, identifying efficacious, safe treatments that address both is an important objective.

One therapy with the potential to address underlying biological processes responsible for OA pathogenesis is platelet-rich plasma (PRP), an autologous blood product that contains an elevated concentration of platelets. Activation of PRP releases an initial burst then a sustained release of growth factors and other molecules, including platelet-derived growth factor, transforming growth factor-β, type I insulin-like growth factor and vascular endothelial growth factor [11]. Animal studies have shown that these proteins are responsible for a range of critical tissue healing roles such as chondrocyte apoptosis inhibition, bone and vessel remodelling, inflammatory modulation, and importantly, collagen synthesis. Additionally, other bioactive molecules released by platelets, such as fibrin, act as a scaffold and chemo-attractant for further migration of stem and other cells to the damaged tissue [11]. Given the limited repair capacity of articular cartilage, these roles offer a mechanism by which PRP may enhance tissue healing and cartilage regeneration in knee OA.

There is some randomised controlled trial (RCT) evidence about the symptomatic effects of PRP for knee OA. A recent systematic review that included 14 RCTs concluded that PRP was likely to be more effective for pain relief and physical function when compared with control injections that included normal saline, HA or glucocorticoids [12]. However, all RCTs were found to be at a moderate to high risk of bias, and most lacked longer-term follow-up and were inadequately powered with small sample sizes. Only three trials compared PRP to placebo, with results showing significantly greater improvements in symptoms over normal saline at six [13] and 12 months [14, 15]. However, all three studies suffered from major methodological flaws including a lack of adequate blinding suggesting the benefits may have been overestimated. No study has investigated the structural effects of PRP.

Heterogeneity in the preparation and injection methods used by published studies has also limited the ability to determine optimal PRP protocols. However, protocol characteristics of those RCTs that have reported positive effects on pain and function have generally utilised a single centrifugation at a slower speed (approximately 1500 g) for approximately 5 min to yield leucocyte poor PRP, and injected fresh PRP (i.e. not frozen and then thawed for subsequent injections) over 3 injections at weekly intervals [16]. There is some preliminary evidence to suggest younger patients, and those with less severe radiographic disease may experience greater symptomatic benefits [14, 17–19].

The primary aim of this two-arm randomised, placebo-controlled trial is to determine if a series of injections of PRP into the knee joint leads to significantly greater reductions in average knee pain severity and less loss of medial tibial cartilage volume when compared to placebo saline injections over 12 months in people with symptomatic knee OA. The secondary aim is to determine if PRP has significantly greater benefits for other clinical (other knee pain measures, physical function, quality of life, participant global rating of change) and magnetic resonance imaging (MRI) structural outcomes (effusion, synovitis, cartilage morphology, bone marrow lesions, cartilage defects, meniscal morphology) compared to placebo injections at 2 (clinical) and 12 (clinical and MRI) months. We will also conduct an embedded economic evaluation of PRP if benefits are found.

## Methods/design

### Trial design

This protocol is described using the 2013 SPIRIT guidelines on standard protocol items for clinical trials [20]. The trial is designed as a two-group, superiority, randomised, participant-, interventionist- and assessor-blinded placebo-controlled trial, to be conducted in Melbourne and Sydney over 4 years. The primary end-point for analysis of outcomes is 12 months after baseline assessment.

### Participants

We will recruit 288 participants aged over 50 years with painful knee OA in one or both knees, and mild to moderately severe structural change on x-rays, from the community via advertisements, print/radio/social media, clinicians and our volunteer database. Participants are eligible for the study if they meet all inclusion criteria below:

i. Aged > 50 years;
ii. knee pain on most days in the last month;
iii. have Kellgren and Lawrence grade 2 or 3 tibiofemoral OA on x-ray; and
iv. report an average level of overall knee pain over the past week of at least 4 on an 11-point numeric rating scale in the target knee.

Participants will be excluded if they:

i. have Kellgren and Lawrence grade 1 (indicating questionable disease) or grade 4 (indicating severe disease);
ii. have lateral joint space narrowing greater than or equal to medial joint space narrowing on x-ray using the Osteoarthritis Research Society International (OARSI) atlas [21];
iii. had injection into the target knee joint of glucocorticoid in the past 3 months or hyaluronic acid in the past 6 months,
iv. had any autologous blood product or stem cell preparation in the past;
v. had knee surgery on their target knee within past 12 months;
vi. have systemic or inflammatory joint disease such as rheumatoid arthritis;
vii. have a history of crystalline or neuropathic arthropathy;
viii. had a knee joint replacement or high tibial osteotomy on their target knee;
ix. plan for joint surgery in the target knee in next 12 months;
x. have other muscular, joint or neurological condition affecting lower limb function;
xi. have a needle phobia;
xii. have immunosuppression or acute infective processes;
xiii. have cancer or other tumours in the last 3 years, or undergone any treatment for cancer or tumours in the last 3 years;
xiv. have a bleeding disorder or are receiving anti-coagulation therapy;
xv. have the presence of a warm, tense joint effusion;
xvi. have a platelet count < 150,000/μL;
xvii. have any other medical condition precluding participation in the study including contraindication to MRI such as a pacemaker;
xviii. pregnancy;
xix. are unwilling to discontinue non-steroidal anti-inflammatory drug and other analgesic usage, with the exception of paracetamol for rescue pain relief, from 2 weeks before the baseline assessment until the 12-month follow-up assessment;
xx. have a body mass index (BMI) > 40 kg/m^2^ because of problems fitting into the MRI machine knee coil;
xxi. unable to attend the six study appointments over 12 months; and
xxii. cannot understand written/spoken English.

### Procedures

**Fig. 1:**
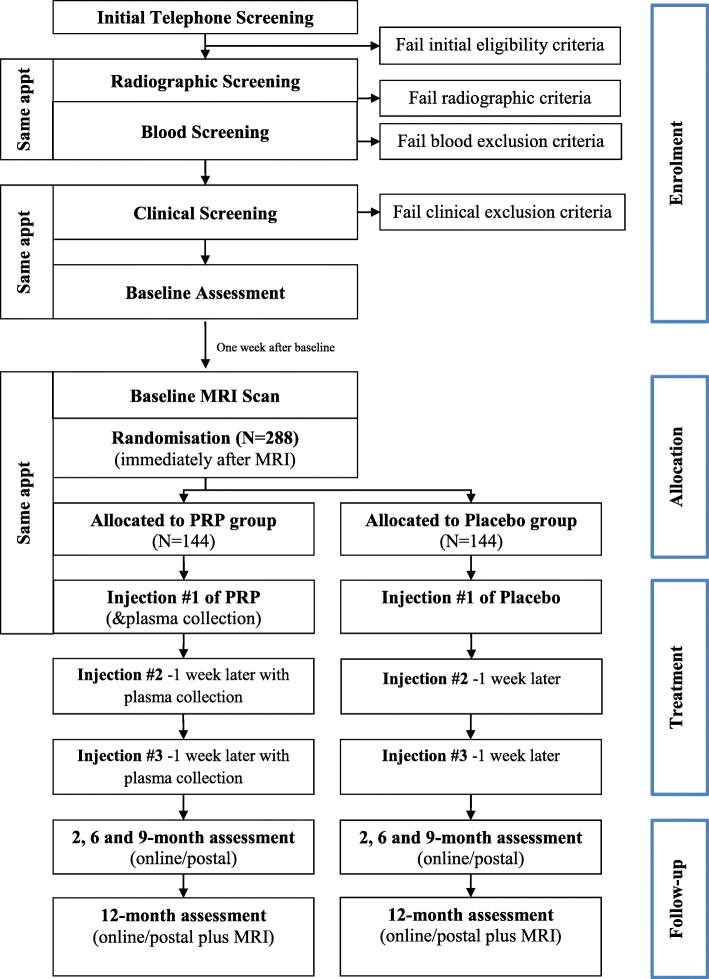
Flow diagram of the study protocol

Figure 1 and Table 1 outline the trial phases and the schedule for enrolment, screening, interventions and assessments. Participants will complete an online survey to determine initial eligibility and will then be screened over the telephone by one of the researchers. Participants deemed suitable from the telephone screening will be invited to undergo standardized bilateral posteroanterior radiographic and blood screening. Participants who pass telephone, radiographic and blood screening will be invited to attend an appointment with the local site coordinator at either the Department of Physiotherapy at the University of Melbourne in Victoria or the Royal North Shore Hospital in New South Wales, Australia. At this visit, the site coordinator will perform a physical assessment of the knee, and participants who do not have a warm, tense knee effusion will then be deemed suitable for the study. Eligible participants will then complete the baseline clinical and health outcome questionnaire, either on paper or electronically on a PC depending on their preference, and will then attend one of the radiology centres for their baseline knee MRI scans. For those participants with bilaterally eligible knees, the most symptomatic knee will be deemed the study knee. Participants will be asked to discontinue non-steroidal anti-inflammatory drugs and other analgesics for knee pain, with the exception of paracetamol for rescue pain relief, from 2 weeks before the baseline assessment until the 12-month follow-up assessment.

**Table 1:** SPIRIT (Standard Protocol Items: Recommendations for Interventional Trials) diagram of enrolment, interventions and assessments for the RESTORE trial

Timepoint			Study period						
	Enrolment	Baseline	Injection 1	Injection 2	Injection 3	Post-allocation			Close-out
	-t_1_		0	1w	2w	2 m	6 m	9 m	12 m
Enrolment:									
Eligibility screen	X								
Informed consent	X								
Allocation		X							
Interventions:									
Platelet rich plasma			X	X	X				
Saline			X	X	X				
Assessments:									
Primary outcomes									
Average overall knee pain severity		X				X	X	X	X
Medial tibial cartilage volume		X							X
Secondary outcomes									
Walking pain severity		X				X			X
KOOS		X							
ICOAP		X							
Participant global ratings of change						X			X
AQoL-8D		X				X	X	X	X
MOAKS sub-scores		X							X
BML and cartilage defects score		X							X
Other measures									
Drug/supplement use		X				X			X
Cytokines and growth factors			X	X	X				
Number of injections			X	X	X				
Adverse events, co-interventions						X			X
Success of blinding					X				
Descriptive measures									
Weight, height, BMI		X							X
Age, gender, symptom duration, x-ray severity, knee alignment, employment, symptom laterality, other symptoms, treatment expectation, PainDETECT, PASE, knee effusion		X							
Health economic data									
QALYs, cost-effectiveness ratio, WHOHPQ		X				X	X	X	X

*ICOAP* Intermittent and Constant Osteoarthritis Pain, *KOOS* Knee Injury and Osteoarthritis Outcome Score, *AQOL-8D* Assessment of Quality of Life Instrument (8D version), *MOAKS* Magnetic resonance imaging OA Knee Score, *BMI* Body mass index, *PASE* Physical Activity Scale for the Elderly, *QALYs* Quality Adjusted Life Years, *WHO HPQ* World Health Organisation Health Performance Questionnaire

Paper-based or electronic clinical and health outcome questionnaires will be sent to participants at 2, 6, 9 and 12 months to complete primary and secondary outcomes at home. Structural outcome measures recorded using MRI of the knee joint will be conducted at baseline and 12 months at radiology practices in Melbourne and Sydney. Ethical approval has been obtained from the University of Melbourne Human Research Ethics Committee (HREC No. 1647671) and the Northern Sydney Local Health Districts Human Research Ethics Committee (HREC/16/HAWKE/430). All participants will provide written informed consent.

#### Randomisation procedures

Participants will be randomly allocated by telephone to the PRP injection group or the placebo injection group using a randomisation schedule prepared and stored by the National Health and Medical Research Council (NHMRC) Clinical Trial Centre. Randomisation will occur according to a 1:1 allocation ratio in blocks with varying sizes of 6 or 10, stratified according to site (Melbourne or Sydney) and radiographic disease severity (Kellgren and Lawrence grades 2 or 3). At the first treatment appointment, a study nurse will telephone the NHMRC Clinical Trials Centre just before administration of the first injection to reveal the participant’s group allocation.

#### Blinding arrangements

Participants will not be told until the end of the study which group they were allocated to. Blood will be withdrawn from the arm of participants in both groups to ensure participant blinding. Blood from participants in the placebo group will be disposed of by the study nurse in a separate location. A study nurse will prepare the injection in a separate room and place a patient label over the syringe and needle base to occlude the contents. The nurse will then give the syringe to the injecting doctor who will not know or be able to tell whether the syringe has PRP or saline. The same preparation process will be in place for injection appointment 2 and 3. All clinical and MRI assessments will be conducted by an assessor blinded to treatment allocation.

#### Interventions

Participants in both the PRP and placebo groups will undergo three intra-articular knee injections performed at weekly intervals, in line with commonly used injection protocols for this condition [16, 22]. The doctor will give the participant the option of having a local anaesthetic injection superficially (subcutaneously) before treatment. The doctor will then check the study knee for the presence of an effusion using ultrasound. If present and deemed to be amenable to aspiration, then this will be performed using a needle inserted in the suprapatellar bursa, and the volume aspirated will be recorded. The study nurse will give the doctor an occluded syringe with a needle containing 5 ml of either normal saline or PRP, and the contents will be injected into the knee joint under ultrasound guidance using a medial patellofemoral approach. Following the injection, passive knee flexion and extension will be performed five times with the participant observed resting for 10 min thereafter.

Participants will return for their second injection approximately 1 week later, and again for a third and final injection approximately 1 week after that. The injection process will be the same as the first injection. After the third injection, participants will be asked about which group they believe they are in, and separately the injecting doctors will also be asked which group they believe the participant is in. After each injection, the study nurse will ask the participant if they experienced any adverse events following the previous injection.

#### PRP preparation

Platelet-rich plasma will be prepared using a commercially-available product (Regen Lab SA), which yields a platelet concentration factor of 1.6 to 5 times over whole blood values, and with approximately 80% platelet recovery [23]. After the blood draw into the Regen blood collection tubes, the tube will be gently inverted several times to mix the anti-coagulant with the blood sample. Samples will then be centrifuged at 1500 g for 5 min in a separate room by the study nurse. After centrifugation, the tubes will be gently agitated to ensure all visible platelet aggregates detach from the separating gel and the tube wall, and 5 mL will be gently withdrawn into a syringe. At the Melbourne site only, a portion of the PRP (approximately 4 mL) will be withdrawn and saved for analysis. A video of the PRP preparation procedure can be found in at https://www.youtube.com/watch?v=Cq8UlbL18Yg&feature=youtu.be.

#### Saline injections

The placebo group will have the blood sample taken as per the PRP group to assist blinding. The research nurse will discard the sample in a separate room, and then prepare a syringe with a saline solution (5 mL), attach a needle and place a patient label over the syringe and needle base to occlude the contents. The nurse will then deliver the syringe to the treating doctor for injection.

### Outcome measures

Study outcome measures are presented in Table 1. Our two primary outcome measures are 12-month change in overall average knee pain severity and medial tibial cartilage volume:

i) *Average overall knee pain severity*: this will be measured at baseline, 2, 6, 9 and 12 months using an 11-point numeric rating scale (NRS) with terminal descriptors ‘no pain’ (score 0) and ‘worst pain possible’ (score 10). This was chosen because it has well-established clinimetric properties in knee OA and established minimal clinically important differences [24], and is a recommended measure for knee OA RCTs from the Osteoarthritis Research Society International [25].
ii) *Medial tibial cartilage volume*: an MRI scan of the study knee will be performed at baseline and 12 months using a 3 T whole body system with a dedicated extremity coil and a T1-weighted fat-suppressed 3D gradient recall acquisition sequence (Additional file 1). Medial tibial cartilage volume will be measured by manually drawing disarticulation contours around the cartilage edges, section by section. To reduce measurement error and bias, one person will measure each participant’s paired set of images, blinded to time sequence and treatment allocation. Regular quality review will occur in 20% of images by a second reader. Our coefficients of variation for these measures are 2.3–2.4% [26].

The following secondary outcome measures will also be collected:

i) *Average knee pain severity during walking*: measured at baseline, 2 months and 12 months on an 11-point NRS, where 0 indicates no pain and 10 indicates worst pain possible.
ii) *Intermittent and Constant Osteoarthritis Pain (ICOAP) questionnaire:* measured at baseline, 2 months and 12 months. This is a self-report multi-dimensional, OA-specific measure of pain experience, with established reliability, internal consistency and validity in people with knee OA [27]. It is comprised of 5-items assessing constant knee pain in the previous week, and 6-items assessing intermittent knee pain in the previous week. Questions regarding pain intensity have terminal responses of ‘not at all’ (score 0) and ‘extremely’ (score 4), and questions regarding pain frequency have ‘never’ (score 0) to ‘very often’ (score 4). Total scores range from 0 to 100, with higher scores indicating worse pain.
iii) *Knee Injury and Osteoarthritis Outcome Score:* measured at baseline, 2 months and 12 months. This is a knee-specific self-report outcome measure with high test-retest reliability, internally consistent and face and content validity [28, 29]. Likert responses range from None to Extreme, and scores range from 0 to 100, with lower scores indicating worse symptoms, function or quality of life. It is comprised of the following five subscales:
  - Pain: scored from 9 questions about knee pain frequency experienced in the last week, and the amount of knee pain experienced during specific activities such as twisting, bending and walking.
  - Other symptoms: scored from 7 questions regarding other symptoms experienced in the last week, such as swelling, restricted range of motion and mechanical symptoms.
  - Function in daily living: scored from 17 questions regarding knee function in the last week.
  - Function in sport and recreation: scored from 5 questions regarding function with sport and recreational activities in the last week.
  - Knee-related quality of life: scored from 4 questions about knee-related quality of life experienced in the last week.
iv) *Participant-perceived global ratings of change:* participants will rate perceived overall change with treatment (compared to baseline), and change in pain and physical function, on a 7-point Likert scale (from “much worse” to “much better”) at 2 and 12 months [30]. Those ‘moderately better’ or ‘much better’ will be classified as improved. All other respondents will be classified as not improved.
v) *Health-related quality of life:* measured at baseline, 2 months and 12 months using the 35-item Assessment of Quality of Life (AQoL-8D) instrument [31]. The AQoL-8D has strong psychometric properties and is more responsive than other widely-used scales [32]. Scores range from − 0.04 to 1.00, with higher scores indicating better quality of life. The AQoL-8D will also be measured at 6 and 9 months for the health economic evaluation.
vi) *Other MRI-derived measurements*: using a 3 T whole body system with a dedicated extremity coil and a T1-weighted fat-suppressed 3D gradient recall acquisition sequence (Additional file 1). Outcomes include:
  - MRI osteoarthritis knee score (MOAKS): an OA-specific semi-quantitative tool evaluating multi-feature joint changes associated with OA [33]. We will assess the following subscores: meniscal morphology (any regions with worsening at 12 months compared to baseline; scored as yes or no); inter-condylar synovitis (worsening in inter-condylar synovitis at 12 months compared to baseline; scored as yes or no); cartilage morphology (number of areas with worsening in thickness at 12 months compared to baseline; categorised as 0, 1, 2, or 3+); and whole knee effusion (change in whole knee effusion at 12 months compared to baseline; categorised as worsened, no change or improved).
  - Bone marrow lesion size: assessed from the MRIs at baseline and 12-months using categorical scoring in the medial distal femur and the proximal tibia (range 0–3 per region, with higher scores indicating greater bone marrow lesion size). Progression of bone marrow lesions (yes/no) will be defined as an increase in score by at least 1 from baseline to follow-up in either the medial tibial or medial femoral compartment.
  - Cartilage defects: score in the medial distal femur and the proximal tibia at baseline and 12-months using categorical scoring (range 0–4 per region, with higher scores indicating greater cartilage defects). Progression of medial cartilage defects (yes/no) will be defined as an increase in score by at least 1 from baseline to follow-up in either the medial tibial or medial femoral compartment.

We will also collect a number of additional measures, as well as data regarding participant descriptive characteristics, health services usage and cytokine and growth factor profiles:

i) *Additional measures*: these include participant weight, drug/supplement use, adverse events, the success of participant and doctor blinding, use of co-interventions and number of injections.
ii) *Descriptive data*: including participant height, body mass index, age, gender, duration of symptoms, radiographic disease severity, knee alignment (measured from the knee x-ray and reported in degrees [34]), current employment, symptom laterality, symptoms in other joints, treatment expectation on a 5 point Likert scale (from “no effect at all” to “complete recovery”), neuropathic pain using the painDETECT questionnaire [35], and physical activity using the Physical Activity Scale for the Elderly (PASE) [36, 37]. The cross-sectional area of the medial tibial plateau from MRI [30] will be measured for use as a covariate.
iii) *Health economic evaluation data*: measured using data collected at baseline, and 2, 6, 9 and 12-months. Data include health care costs estimated from healthcare service usage and Quality Adjusted Life Years (QALYs) calculated using the AQoL-8D, and work performance data obtained from the World Health Organisation Health Performance Questionnaire (WHO HPQ) [38].
iv) *Cytokine and growth factor concentrations:* aliquots of PRP will be analysed in a subset of participants to determine the concentration of key growth factors and cytokines, such as platelet-derived growth factor, transforming growth factor beta 1, connective tissue growth factor, interleukin 1 receptor agonist, interleukin 1 beta, interleukin 6 and matrix metallopeptidase 9.

### Participant compliance

Compliance will be reported as the number of injections administered. Compliance with the injection schedule will be monitored by the site coordinators and the staff at the radiology clinics, who will be responsible for booking participants in for injection procedures.

### Adverse effects of treatment and co-interventions

Adverse events are defined as any untoward medical occurrence in a trial participant which does not necessarily have a causal relationship with the treatment. Adverse events will be collected via self-report during the 2- and 12-month data collection, and by the study nurse following each injection. This will include questions about any adverse events that participants believe may be related to the study intervention, including their nature, how long they lasted for and what action if any, they took (e.g. taking medication or seeing a health professional). Serious adverse events are defined as any untoward and unexpected medical occurrence that results in death, is life-threatening, requires hospitalisation or prolongation of existing inpatient’s hospitalisation, results in persistent or significant disability or incapacity, is a congenital anomaly or birth defect, or any other important medical condition which may require medical or surgical intervention to prevent one of the outcomes listed [39]. Use of co-interventions (medications for knee pain and any other treatments for knee OA) will be recorded at 2 and 12-months.

### Sample size calculations

Primary outcomes will be the 12-month change in (i) overall average knee pain severity over the last week measured using an 11-point numeric rating scale; and (ii) medial tibial cartilage volume from MRI expressed as a percentage. The minimum clinically important difference to be detected in OA trials is a change in pain of 1.8 units (out of 10) [24]. Using control group data from our 12-month RCT in people with mild to moderate knee OA, we assume a between-participant standard deviation (SD) of change in pain of 2.4 and baseline to 12-month correlation in scores of 0.29 [30]. We wish to find a 40% reduction in the amount of medial tibial cartilage volume loss with PRP. Based on our data [40], slowing the rate of cartilage loss by this amount could delay the need for knee replacement. We anticipate the control group to lose 2.8% (SD 3.5%) of medial tibial cartilage volume in 12-months [30], with a conservative baseline to 12-month correlation in scores of 0.50. These assumptions, together with an analysis of covariance adjusted for baseline scores, indicate that 115 participants per arm will have 80% power to detect a 40% reduction in loss of cartilage volume with a two-sided significance level of 0.05. With this number of participants, we also have > 99% power to detect the minimum clinically important difference in pain. Allowing for 20% loss to follow-up, we will recruit 144 participants per arm - total 288 participants.

### Statistical analyses

#### Primary analyses

We will conduct an intention-to-treat analysis whereby all participants will be included in the study in the group to which they were randomised. Analysis will be conducted by a biostatistician blinded to treatment group, with two-sided hypothesis tests and *p*-values < 0.05 significant. If the proportion of missing data exceeds 5%, missing outcome data will be imputed using multiple imputation methodology, and sensitivity to the missing at random assumption will be investigated [41]. Changes from baseline will be presented for each group at 2- and 12-month time points using the mean change and 95% confidence intervals. For continuous outcomes (e.g. pain, cartilage volume, physical function), longitudinal analyses will be conducted, with differences in mean change (follow-up minus baseline) compared between the groups using mixed linear regression models with the baseline value, stratifying variables (Kellgren and Lawrence grade and injecting site) and an interaction between month and treatment group as covariates, including random effects for participants [42]. Models including baseline cartilage volume, age, gender, body mass index and cross-sectional area of the medial tibial plateau from MRI [30] will also be fit. Appropriate transformations of outcome measures will be considered if needed to meet statistical assumptions (e.g. linearity, normality and homogeneity of residuals) as assessed using diagnostic plots. Binary outcomes will be compared between groups using risk differences calculated after fitting longitudinal regression models for binary outcomes, adjusted for stratification variables and accounting for clustering of measurements within participants. The other MRI-derived measurements (MOAKS, bone marrow lesion size and cartilage defects) will be compared between groups using appropriate models, adjusting for age, gender, body mass index, and the stratifying variables. The model for cartilage defects will also be adjusted for cross-sectional area of the medial tibial plateau [30]. If appropriate, the effect of PRP on primary outcomes under hypothetical full adherence to assigned treatment will be investigated. The success of blinding will be assessed using the James Blinding Index [43].

#### Moderator analyses

We will also conduct planned exploratory analyses to investigate potential moderators that could influence response to treatment at 12 months. Pre-identified potential moderators include KL grade, body mass index, knee effusion on MRI and knee alignment. A description of the hypothesis for each moderator analysis and the rationale is found in Additional file 2. To assess the moderation of the effect of randomised treatment group by binary potential moderators (KL grade and effusion), an interaction term between randomised group and the potential moderator, as well as terms for the randomised group and the potential moderator, will be included in outcome regression models. To assess the moderation of the effect of randomised treatment group by continuous potential moderators (body mass index and knee alignment), the multivariable fractional polynomial interaction approach described previously [44] will be applied. This approach allows for nonlinear functional forms of the continuous potential moderator to be included in the regression model for outcomes, with the potential for separate nonlinear functional forms in each treatment group.

#### Economic analyses

The cost-effectiveness of PRP will be estimated by calculating the incremental average health care cost of those treated and the difference in pain score and health-related quality of life over the 12 months of the trial compared to the placebo group. The comparison will be reported as the incremental cost per unit change in the pain score, the incremental cost per additional quality-adjusted life year, and the net benefit of treatment at 12 months. The quality-adjusted life year will be calculated using the average AQoL-8D value. Net benefits will be calculated using a range of potential money values of a quality-adjusted life year. Costs will include the cost of treatment and associated imaging as well as the downstream medical, pharmaceutical and hospital costs in each arm. Health care utilisation data will be collected by questionnaire. Cost-effectiveness will be calculated using separate generalised linear regression models for costs and outcomes controlling for baseline levels. Predicted costs and outcomes will be used to calculate means for cost-effectiveness ratios and net benefits with bootstrapped 95% confidence intervals. If there is no demonstrated benefit from PRP, we will calculate the incremental average health care cost of those treated. In the secondary economic analysis, the impact on employment and productivity at work will be calculated from data collected by questionnaire.

### Timelines

Ethics approval was obtained from the Human Research Ethics Committee of the University of Melbourne in October 2016. Ethics approval was obtained from the North Sydney Local Health Districts ethics committee in March 2017 and governance approval from the Northern Sydney Local Health Districts Governance office in July 2017. Recruitment commenced in August 2017 and is anticipated to be completed in December 2019. The trial is due for completion in December 2020 when all participants will have completed 12-month follow-up.

## Discussion

Identifying treatments that reduce symptoms and slow disease progression in knee OA is an important OA research objective. Outcomes from this study will provide the first high-quality RCT evidence of the symptomatic and structural benefits of PRP to either support or discourage use of PRP for knee OA. This is important given that several systematic reviews [22, 45, 46] have highlighted the limited number of RCTs that included a placebo control, all previous trials have a moderate to high risk of bias, and none have included structural outcomes. Likewise, current clinical guidelines vary in their recommendation for PRP, with some not including recommendations either for or against PRP given the lack of evidence [47, 48], and others recommending against their use [4]. Findings from the RESTORE trial will, therefore, provide essential information to fill a major evidence gap in the literature and will inform international clinical practice guidelines.

## Additional files

Additional file 1: Proposed sequences for RESTORE knee study using knee coil. Knee MRI sequences and specifications for the RESTORE trial. (DOCX 18 kb)

Additional file 2: Potential moderators of PRP treatment effects, and their associated hypotheses and rationale for inclusion. Potential moderators of PRP treatment effects, associated hypotheses and rationale for inclusion for RESTORE trial. (DOCX 28 kb)

## Abbreviations

AQoL: Assessment of Quality of Life
HREC: Human Research Ethics Committee
ICOAP: Intermittent and Constant Osteoarthritis Pain questionnaire
KOOS: Knee Injury and Osteoarthritis Outcome Score
MOAKS: Magnetic resonance imaging Osteoarthritis Knee Score
MRI: Magnetic resonance imaging
NHMRC: National Health and Medical Research Council
NRS: Numerical rating scale
OA: Osteoarthritis
PASE: Physical Activity Scale for the Elderly
PRP: Platelet-rich plasma
QALYs: Quality Adjusted Life Years
RCT: Randomised controlled trial
WHO HPQ: World Health Organisation Health Performance Questionnaire
