# Supplementary material for: Efficacy of intra-articular injections of platelet-rich plasma as a symptom- and disease-modifying treatment for knee osteoarthritis - the RESTORE trial protocol
Source: BMC Musculoskelet Disord. 2018 Jul 28;19:272. doi: 10.1186/s12891-018-2205-5 (PMC6064619; doi:10.1186/s12891-018-2205-5)
Supplement: Supplementary file 1 — Proposed sequences for RESTORE knee study using knee coil. Knee MRI sequences and specifications for the RESTORE trial. (DOCX 18 kb) [file 12891_2018_2205_MOESM1_ESM.docx]

**Platelet-rich plasma as a symptom- and disease-modifying treatment for knee osteoarthritis - the RESTORE trial** ***protocol***

**Additional file 1**

Table 1. Proposed sequences for RESTORE knee study using knee coil.

| **Sequence** | **Slices** | **Slice thickness (mm)** | **Slice Gap (mm)** | **Phase Encoding** | **Scan time** | **Ipat** | **Resolution** | **Turbo Factor (TSE)** | **Voxel size** | **TR** | **TE** | **Averages (NSA)** | **Bandwidth** | **Fat Sat** |
| --- | --- | --- | --- | --- | --- | --- | --- | --- | --- | --- | --- | --- | --- | --- |
| PD FS Sag | 40 | 2.2 | 0.2 | H>F | 2.36 | 2 | 307x384 | 7 | 0.4x0.4x2.2 | 3500 | 38 | 2 | 200 | Yes |
| Ax PD FS | 40 | 2.5 | 0.3 | R>L | 3.12 | 2 | 384x278 | 7 | 0.4x0.4x2.5 | 4170 | 30 | 2 | 221 | Yes |
| PD Cor | 40 | 2.5 | 0.3 | R>L | 1.59 | 2 | 358x448 | 7 | 0.3x0.3x2.5 | 3300 | 38 | 1 | 222 | No |
| PD FS Cor | 40 | 2.5 | 0.3 | R>L | 1.59 | 2 | 307x384 | 7 | 0.4x0.4x2.5 | 3600 | 36 | 1 | 224 | Yes |
| T1 3D Gradient DESS Sag | 192 | 0.6 |  | A>P | 6.32 | 2 | 265  95%phase  90%slice | - | Acq & Rec 0.66x0.63x0.66 | 14.10 | 5 | 2 | 250 | Yes |
