# Supplementary material for: Efficacy of intra-articular injections of platelet-rich plasma as a symptom- and disease-modifying treatment for knee osteoarthritis - the RESTORE trial protocol
Source: BMC Musculoskelet Disord. 2018 Jul 28;19:272. doi: 10.1186/s12891-018-2205-5 (PMC6064619; doi:10.1186/s12891-018-2205-5)
Supplement: Supplementary file 2 — Potential moderators of PRP treatment effects, and their associated hypotheses and rationale for inclusion. Potential moderators of PRP treatment effects, associated hypotheses and rationale for inclusion for RESTORE trial. (DOCX 28 kb) [file 12891_2018_2205_MOESM2_ESM.docx]

**Platelet-rich plasma as a symptom- and disease-modifying treatment for knee osteoarthritis - the RESTORE trial** **protocol**

**Additional file 2**

Table 1: Potential moderators of PRP treatment effects, and their associated hypotheses and rationale for inclusion.

|  | **Variable** | **Hypotheses** | **Justification** |
| --- | --- | --- | --- |
|  | KL grade (2 or 3) | - The symptomatic and structural benefits of PRP will be greater in those with KL2 compared to KL3. | - Higher KL grade indicates greater structural damage, therefore the symptomatic and structural benefits of PRP may be less in those with greater disease severity. - Multiple (3) injections of PRP have been shown to achieve greater efficacy in patients with early OA whereas they did not significantly improve pain and other symptoms in patients with advanced OA [1]. |
|  | BMI | - The symptomatic and structural benefits of PRP will be greater in those with a lower BMI compared to those with a higher BMI. | - Knee OA-related pain and structural damage in people who are obese may also have a mechanical origin, and this is likely to continue after the injections and throughout the follow up period. Therefore, these patients may be less likely to respond to PRP injections. - Younger and more active patients are likely to have a lower BMI, and these patients (with cartilage lesions or early OA) achieved better changes in pain and function [2]. |
|  | Effusion (present/absent) | - The symptomatic and structural benefits of PRP will be greater in those without a knee joint effusion compared to those with a knee joint effusion. | - A knee joint effusion will contain a higher concentration of pro-inflammatory cytokines therefore PRP may be less effective in these patients. |
|  | Static knee alignment | - The symptomatic and structural benefits of PRP will be greater in those with less varus knee malalignment than in those with more knee malalignment. | - A varus knee malalignment has been shown to increase knee loads [3], and higher knee loads are associated with worse knee OA symptoms [4, 5] and structural progression [4, 6-9]. Given the malalignment will remain after the injections, this could interfere with any benefits of PRP. |

**REFERENCES**

1. Görmeli G, Görmeli CA, Ataoglu B, Çolak C, Aslantürk O, Ertem K. Multiple PRP injections are more effective than single injections and hyaluronic acid in knees with early osteoarthritis: a randomized, double-blind, placebo-controlled trial. Knee Surgery, Sports Traumatology, Arthroscopy. 2015:1-8.

2. Kon E, Mandelbaum B, Buda R, Filardo G, Delcogliano M, Timoncini A, Fornasari PM, Giannini S, Marcacci M. Platelet-rich plasma intra-articular injection versus hyaluronic acid viscosupplementation as treatments for cartilage pathology: From early degeneration to osteoarthritis. Arthroscopy: The Journal of Arthroscopic & Related Surgery. 2011; 27:1490-1501.

3. Maneekittichot T, Sorachaimetha P, Onmanee P, Chanthasopeephan T: The effect of vary varus malalignment on knee adduction moment during walking of human normal gait. In: *2013 35th Annual International Conference of the IEEE Engineering in Medicine and Biology Society (EMBC): 2013*: IEEE; 2013: 7229-7232.

4. Miyazaki T, Wada M, Kawahara H, Sato M, Baba H, Shimada S. Dynamic load at baseline can predict radiographic disease progression in medial compartment knee osteoarthritis. Annals of the Rheumatic Diseases. 2002; 61:617-622.

5. Kito N, Shinkoda K, Yamasaki T, Kanemura N, Anan M, Okanishi N, Ozawa J, Moriyama H. Contribution of knee adduction moment impulse to pain and disability in Japanese women with medial knee osteoarthritis. Clinical Biomechanics. 2010; 25:914-919.

6. Chehab EF, Favre J, Erhart-Hledik JC, Andriacchi TP. Baseline knee adduction and flexion moments during walking are both associated with 5 year cartilage changes in patients with medial knee osteoarthritis. Osteoarthritis and Cartilage. 2014; 22:1833-1839.

7. Bennell KL, Bowles K-A, Wang Y, Cicuttini F, Davies-Tuck M, Hinman RS. Higher dynamic medial knee load predicts greater cartilage loss over 12 months in medial knee osteoarthritis. Annals of the Rheumatic Diseases. 2011; 70:1770-1774.

8. Maly MR, Acker SM, Totterman S, Tamez-Peña J, Stratford PW, Callaghan JP, Adachi JD, Beattie KA. Knee adduction moment relates to medial femoral and tibial cartilage morphology in clinical knee osteoarthritis. Journal of Biomechanics. 2015; 48:3495-3501.

9. Chang AH, Moisio KC, Chmiel JS, Eckstein F, Guermazi A, Prasad PV, Zhang Y, Almagor O, Belisle L, Hayes K *et al*. External knee adduction and flexion moments during gait and medial tibiofemoral disease progression in knee osteoarthritis. Osteoarthritis and Cartilage. 2015; 23:1099-1106.
